# Effective Analgesia with Bilateral Erector Spinae Plane Catheters for a Patient with Traumatic Rib and Spine Fractures

**DOI:** 10.1155/2019/9159878

**Published:** 2019-04-08

**Authors:** L. Klesius, K. Schroeder

**Affiliations:** Department of Anesthesiology, University of Wisconsin-Madison, Madison, WI, USA

## Abstract

Pain management in trauma patients with acute rib and spine fractures presents a challenge for the anesthesiologist and achieving adequate analgesia is important in preventing pulmonary complications. Unfortunately, neuraxial techniques are often challenging or contraindicated due to spine fractures or coagulopathy. Erector spinae plane (ESP) blocks provide an alternative regional anesthetic technique to manage pain. We describe a case of bilateral ESP catheters placed intraoperatively after spinal instrumentation in a patient with bilateral rib and spine fractures sustained in a tractor rollover crash. Prior to surgery, the patient had inadequate pain control and poor respiratory function despite multimodal analgesia. With the addition of bilateral ESP catheters, the patient's pain control improved and he was weaned from respiratory support. ESP blocks have been shown to provide effective analgesia in patients with rib fractures; however, the utilization of these blocks has not been described in patients with spine fractures undergoing spinal instrumentation. Thus, ESP blocks provide a simple alternative to providing surgical and trauma analgesia when neuraxial techniques are contraindicated. The success of bilateral ESP catheters in our patient indicates a further area for application of ESP blocks in patients undergoing spine surgery with acute traumatic spine fractures.

## 1. Introduction

Introduced in 2016, the ESP block is a simple fascial plane block with an expanding application in regional anesthesia. Previously, the ESP block has been described in treating acute rib fracture pain, acute and chronic thoracic pain, and more recently for abdominal and lumbar analgesia [1–5]. Although initial studies of the ESP block demonstrated spread of local anesthetic along the thoracolumbar fascia and into the paravertebral space to provide analgesia of both the dorsal and ventral rami of the spinal roots [1], recent cadaveric studies are conflicting regarding potential paravertebral spread [6, 7]. Despite disagreement in the mechanism of ESP blocks and potential limitations in paravertebral spread, a retrospective cohort study published by Adhikary et al. in 2019 demonstrated statistically significant increases in inspiratory spirometry volumes and improved pain scores compared to baseline in patients with multiple rib fractures [8]. This conveys a clinical benefit for the application of ESP blocks in patients with multiple rib fractures based on improvement in respiratory function and analgesia. In addition, application of the ESP block for patients with acute traumatic spine fractures or patients undergoing surgical spinal instrumentation is likely to provide effective analgesia through blockade of the dorsal rami. We report a case of effective analgesia with bilateral ESP catheters for a patient with acute traumatic rib and spine fractures that underwent surgical spinal instrumentation in whom multimodal analgesia was ineffective.

## 2. Case Report

The patient provided written consent for publication.

A 68-year-old man sustained multiple rib and spine fractures after being involved in a tractor rollover crash. His injuries included left 1st-12th rib fractures, right 1st-6th rib fractures, displaced T12 spinous process fracture, L1 burst fracture, L2, 3, and 5 transverse process fractures, and a left clavicle fracture. Due to the extent of his injuries, the patient described severe pain in his thorax and back. The patient was initially treated with multimodal analgesia including scheduled acetaminophen (1000 mg 4 times daily), gabapentin (300 mg 3 times daily), hydromorphone patient-controlled analgesia (PCA) with an average 24-hour hydromorphone consumption of 4.7 mg/day, and lidocaine patches. Given his traumatic spine fractures, an epidural was contraindicated. However, pain remained poorly controlled. A 48-hour continuous infusion of ketamine at 10 mg/hr resulted in a mild improvement in pain control, with ratings decreasing from 10/10 to 8/10 on the numerical rating scale (NRS) for pain. However, the patient continued to require high-flow nasal cannula (HFNC) and intermittent continuous positive airway pressure (CPAP) to improve respiratory function due to splinting from pain.

On postinjury day 3, the patient then presented for surgical repair of his L1 burst fracture with T11-L3 posterior spinal instrumentation. Intraoperative epidural placement by the surgical team was considered, but determined unsafe given the high risk of surgical-site bleeding and concern for epidural hematoma formation. Thus, the decision was made to proceed with intraoperative placement of bilateral ESP catheters.

Following completion of the surgical procedure, the patient was maintained under general anesthesia in the prone position on the operating room table. After informed consent, a right-sided ESP block was performed using ultrasound guidance with a high-frequency linear ultrasound probe. The right T5 transverse process was identified with the ultrasound positioned in a parasagittal orientation approximately 3 cm off midline. A 17-gauge Tuohy needle was advanced in a cephalad to caudad direction using an in-plane needling technique to a position immediately posterior to the T5 transverse process and anterior to the erector spinae muscle. The ESP was identified with a bolus injection of 15 mL of 0.25% bupivacaine with 2.5 mcg/mL of epinephrine into the fascial plane. Then, an indwelling catheter was advanced 5 cm into the ESP. Catheter placement was confirmed with an additional 10 ml of 0.25% bupivacaine with 2.5 mcg/mL of epinephrine injected through the catheter under ultrasound visualization. After securing the catheter, the same procedure was repeated on the left side. Supplemental intraoperative analgesics administered prior to ESP catheter placement included continuation of the ketamine infusion at 10 mg/hr and 100 mcg of fentanyl. No additional analgesics were given intraoperatively.

Postoperatively, analgesia was maintained through the ESP catheters using a programmed intermittent bolus technique with 10 mL of 0.2% ropivacaine injected into each catheter via an infusion pump every 60 minutes in addition to a multimodal analgesia regimen. In the postanesthesia care unit (PACU), the patient initially reported 0/10 pain on the NRS pain scale, with a highest rating of 5/10 at the time of discharge from the PACU. The majority of the pain described by the patient involved anterior rib pain and left shoulder pain; back pain was a smaller contributor to his overall pain burden. The ketamine infusion was discontinued in the PACU given his improvement in pain control. Over the subsequent 7 postoperative days, the patient continued to report significant improvement in pain control in his ribs and back. NRS pain ratings were consistently less than preoperative ratings with scores ranging from 1/10 to 5/10. In addition, the patient localized the most severe pain to his left shoulder, secondary to the clavicle fracture sustained in the trauma. Average 24-hour hydromorphone consumption also decreased to 3.8 mg/day, which was a 20% decrease from preoperative consumption. The modest reduction in opioid consumption was most likely related to the extent of his injuries and his concomitant clavicle fracture. Respiratory status improved and he was successfully weaned from HFNC on postoperative day 1. The ESP catheters were left in situ for 7 days until pain control could be optimized with an oral analgesia regimen and the patient was successfully discharged home.

## 3. Discussion

After introduction of the ESP block in 2016, the application of the block has expanded to include treatment of multiple acute and chronic pain pathologies of the thorax and abdomen [1–4]. While the ESP block has previously been described for alleviation of pain from rib fractures [2, 8], facilitating pain relief after surgical fixation of acute spine fractures has not been described. Similarly, the ESP block has not been previously described in the acute trauma patient with spine fractures as an alternative to neuraxial techniques. The projected cephalad and caudad spread along the thoracolumbar fascia encompassing the dorsal rami of the spinal nerves provides a logical explanation for the analgesic benefit for patients undergoing spine surgery or with acute traumatic spine fractures [1, 4]. There is one case series that describes the successful application of ESP blocks/catheters in spine surgery patients, but this was in the absence of additional injuries related to trauma and placement of the ESP blocks occurred at low thoracic levels near the surgical site [5]. Like other fascial plane blocks, the ESP block is technically straightforward to perform with presumed lower risks of complications including nerve trauma, pneumothorax, or hematoma formation [2, 3]. In trauma patients with intracranial pathology or potential spinal cord injury, the ESP block also allows for continued neurologic examination, which can be confounded when neuraxial techniques are used. In addition, there are fewer contraindications to placement compared to neuraxial techniques, making ESP blocks a potential alternative to neuraxial or even paravertebral techniques. Expanding the application of the ESP block for patients undergoing spine surgeries or with acute traumatic spine fractures could provide an analgesic option for patients previously not considered block candidates. This affords an opportunity for a new area of investigation and research.

## References

[1] Forero M., Adhikary S. D., Lopez H., Tsui C., Chin K. J. (2016). The erector spinae plane block a novel analgesic technique in thoracic neuropathic pain. *Regional Anesthesia and Pain Medicine*.

[2] Hamilton D. L., Manickam B. (2017). Erector spinae plane block for pain relief in rib fractures. *British Journal of Anaesthesia*.

[3] Forero M., Rajarathinam M., Adhikary S., Chin K. J. (2017). Erector spinae plane (ESP) block in the management of post thoracotomy pain syndrome: a case series. *Scandinavian Journal of Pain*.

[4] Chin K. J., Malhas L., Perlas A. (2017). The erector spinae plane block provides visceral abdominal analgesia in bariatric surgery: a report of 3 cases. *Regional Anesthesia and Pain Medicine*.

[5] Melvin J. P., Schrot R. J., Chu G. M., Chin K. J. (2018). Low thoracic erector spinae plane block for perioperative analgesia in lumbosacral spine surgery: a case series. *Canadian Journal of Anesthesia*.

[6] Ivanusic J., Konishi Y., Barrington M. J. (2018). A cadaveric study investigating the mechanism of action of erector spinae blockade. *Regional Anesthesia and Pain Medicine*.

[7] Adhikary S. D., Bernard S., Lopez H., Chin K. J. (2018). Erector spinae plane block versus retrolaminar block: a magnetic resonance imaging and anatomical study. *Regional Anesthesia and Pain Medicine*.

[8] Adhikary S. D., Liu W. M., Fuller E., Cruz-Eng H., Chin K. J. (2019). The effect of erector spinae plane block on respiratory and analgesic outcomes in multiple rib fractures: a retrospective cohort study. *Anaesthesia*.

